# Mapping of B-cell epitopes on the N- terminal and C-terminal segment of nucleocapsid protein from Crimean-Congo hemorrhagic fever virus

**DOI:** 10.1371/journal.pone.0204264

**Published:** 2018-09-20

**Authors:** Abulimiti Moming, Daerken Tuoken, Xihong Yue, Wanxiang Xu, Rong Guo, Dongliang Liu, Yijie Li, Zhihong Hu, Fei Deng, Yujiang Zhang, Surong Sun

**Affiliations:** 1 Xinjiang Key Laboratory of Biological Resources and Genetic Engineering, College of Life Science and Technology, Xinjiang University, Urumqi, China; 2 Center for Disease Control and Prevention of Xinjiang Uygur Autonomous Region, Urumqi, China; 3 Key Lab of Reproduction Regulation of NPFPC, Shanghai Institute of Planned Parenthood Research, Fudan University, Shanghai, China; 4 State Key Laboratory of Virology, Wuhan Institute of Virology, Chinese Academy of Sciences, Wuhan, China; Instituto Butantan, BRAZIL

## Abstract

Crimean-Congo hemorrhagic fever virus (CCHFV) is a tick-borne pathogen that causes severe disease in humans. CCHFV is widely distributed in more than 30 countries and distinct regions, which means that it poses a serious threat to human health. The nucleocapsid protein (NP) encoded by the CCHFV S gene is the primary detectable antigen in infected cells, which makes it an important viral antigen and a clinical diagnostic target. In this study, the modified biosynthetic peptide (BSP) method was used to identify the fine epitopes on the N- and C- terminals of NP from the CCHFV YL04057 strain using rabbit antiserum against CCHFV-NP. Nine epitopes were identified: E1a (^178^NLILNRGG^185^), E1b (^184^GGDENP^189^), E2 (^352^PLKWGKK^358^), E3 (^363^FADDS^367^), E4 (^399^NPDDAA^404^), E5a (^447^DIVASEHL^454^), E5b (^452^EHLLHQSL^459^), E6 (^464^SPFQNAY^470^) and E7 (^475^NATSANII^482^). Western blotting analysis showed that each epitope interacted with the positive serum of sheep that had been naturally infected with CCHFV. Amino acid sequence alignment between each epitope and their homologous proteins showed that they were almost 100% conserved among 12 CCHFV sequences from different lineages, except for epitopes E1a, E1b and E2. Three-dimensional structural modeling analysis showed that all identified epitopes were located on the surface of the NP “head” domain. This study identified fine epitopes on the N- and C- terminals of NP, which will increase the understanding of the structure and function of NP, and it could lay the foundation for the design and development of a CCHFV multi-epitope peptide vaccine and detection antigen.

## Introduction

Crimean-Congo hemorrhagic fever (CCHF) is a tick-borne zoonotic disease that was first discovered in the West Crimean Peninsula in 1944. It has been reported in more than 30 countries and distinct regions in Central and Southern Africa, Southeast Asia, Southeast Europe and the Middle East, with case fatality rates of up to 30–50% [1–3]. In 1965, the first case of CCHF in China was found in Bachu County in Xinjiang [4]. CCHF is now an important insect-borne disease in China, and it is mainly distributed in Xinjiang, a western region of China. Humans are generally infected through tick bites, direct contact with blood or tissue of infected livestock, or nosocomial infections [1, 5, 6]. CCHF is characterized by rapid spread and high mortality. Handling of the infectious virus requires biosafety level 4 (BSL4) containment [7, 8]. At present, there is no effective preventive vaccine or specific antiviral therapy for CCHFV.

CCHFV belongs to the genus *Orthonairovirus* in the family of *Nairoviridae* [9]. The genome consists of three negative-stranded RNAs, designated large (L), medium (M) and small (S), which encode RNA polymerase, glycoprotein precursor (GP) and nucleocapsid protein (NP), respectively [10]. As the predominant protein of CCHFV, NP assembles the viral nucleic acid to form a nucleoprotein known as ribonucleoprotein, and it assists in initiating viral genome replication and mRNA transcription, thus it is an indispensable protein in the CCHFV life cycle [11–14].

The NP has a unique "head" and "stem" structure that forms a racket shape; the N- and C- terminals are located in the "head" domain and are involved in the RNA binding sites [10]. NP has been increasingly regarded as an important target for clinical diagnosis of CCHFV infection and has been shown to be the primary viral antigen that elicits early humoral immunity, being the only protein that can be detected in the early stages of CCHFV infection [15–18]. Therefore, identification of IgG-recognized B-cell epitopes (BCEs) on CCHFV-NP will provide a major molecular basis for the development of a possible vaccine and the detection of viral infection.

In the previous study, five epitopes on the middle region [amino acid (aa) 237–305] of the NP “stem” domain (NP2) were identified using rabbit serum against recombinant (r)-NP combined with the biosynthetic peptide (BSP) method [19]. However, there have only been a few reports on epitope identification in the N- and C- terminal regions (NP1, aa 1–200; NP3, aa 286–482) of CCHFV-NP. In this study, we mapped epitopes in these regions of CCHFV-NP1 and -NP3 using rabbit polyclonal antibodies (pAbs) against CCHFV-NP and an improved glutathione S-transferase (GST)188-BSP method [20, 21]. All the epitopes could be recognized by the antisera of sheep infected with CCHFV. We also analyzed the conservation of each epitope among homologous CCHFV proteins, and their positions in the predicted three-dimensional (3D) structure of NP. These results help us to understand more about epitope distribution on CCHFV-NP, and they provide a solid foundation for the design and development of a preventative CCHFV multi-epitope peptide vaccine and a detection antigen.

## Materials and methods

### Ethics statement

The study was approved by the Committee on the Ethics of Animal Experiments of Xinjiang Key Laboratory of Biological Resources and Genetic Engineering (BRGE-AE001), Xinjiang University. The animal serum samples were collected using random sampling and this process did not involving killing the animals.

### Plasmids and antibodies

pET-32a-*NP1* (aa 1–200), pGEX-KG-*NP2* (aa 170–305) and pET-32a-*NP3* (aa 286–482) were previously constructed and stored by this research group [22]. The prokaryotic expression plasmid pXXGST-3 was donated by Professor Wan-xiang Xu from Shanghai Institute of Planned Parenthood Research. The rabbit pAbs against CCHFV-NP were donated by Professor Fei Deng from Wuhan Institute of Virology, Chinese Academy of Sciences. The sheep serum samples used in the study were collected in 2005 in Bachu County and kindly provided by Professor Yujiang Zhang from Xinjiang Centers for Disease Control and Prevention [23]. The serum sample of CCHFV-infected sheep was previously identified as positive using an immunofluorescent assay (IFA) and indirect reverse transcription polymerase chain reaction (RT-PCR) [23]. In our previous study, we identified the antigenicity of epitopes on NP (237–305) using the same sera of sheep naturally infected with CCHFV [19]. Serum samples of a healthy rabbit and a healthy sheep with no history of CCHFV infection were used as negative controls in the western blotting assay. *Escherichia coli* BL21 (DE3) competent cells were used to express 16/8mer peptides fused with a truncated GST188 protein (i.e., with the initial 188 aa of GST) [24]. Goat anti-rabbit and mouse anti-goat IgG conjugated to horseradish peroxidase (HRP) were purchased from Beijing TransGen Biotech, Co., Ltd. (China).

### Other reagents and materials

DNA ligase and restriction enzymes *Bam*H I and *Sal* I (Takara Co., Ltd, Dalian, China), *E*. *coli* strain BL21 (DE3) competent cells (Novagen, Inc., Madison, USA), QIAquick Gel Extraction Kit (QIAGEN, Duesseldorf, Germany), unstained or prestained molecular weight markers (ThermoFisher Science, Waltham, MA, USA), 0.2 μm nitrocellulose membrane (Whatman GmbH, Dossel, Germany), and enhanced chemiluminescence (ECL) plus western blotting detection kit (GE Healthcare, Buckinghamshire, UK) were obtained. Other general chemicals were obtained from Shanghai Sangon Co., Ltd (China).

#### Mapping strategy and biosynthesis of overlapping 16mer and 8mer peptides

**Fig 1:**
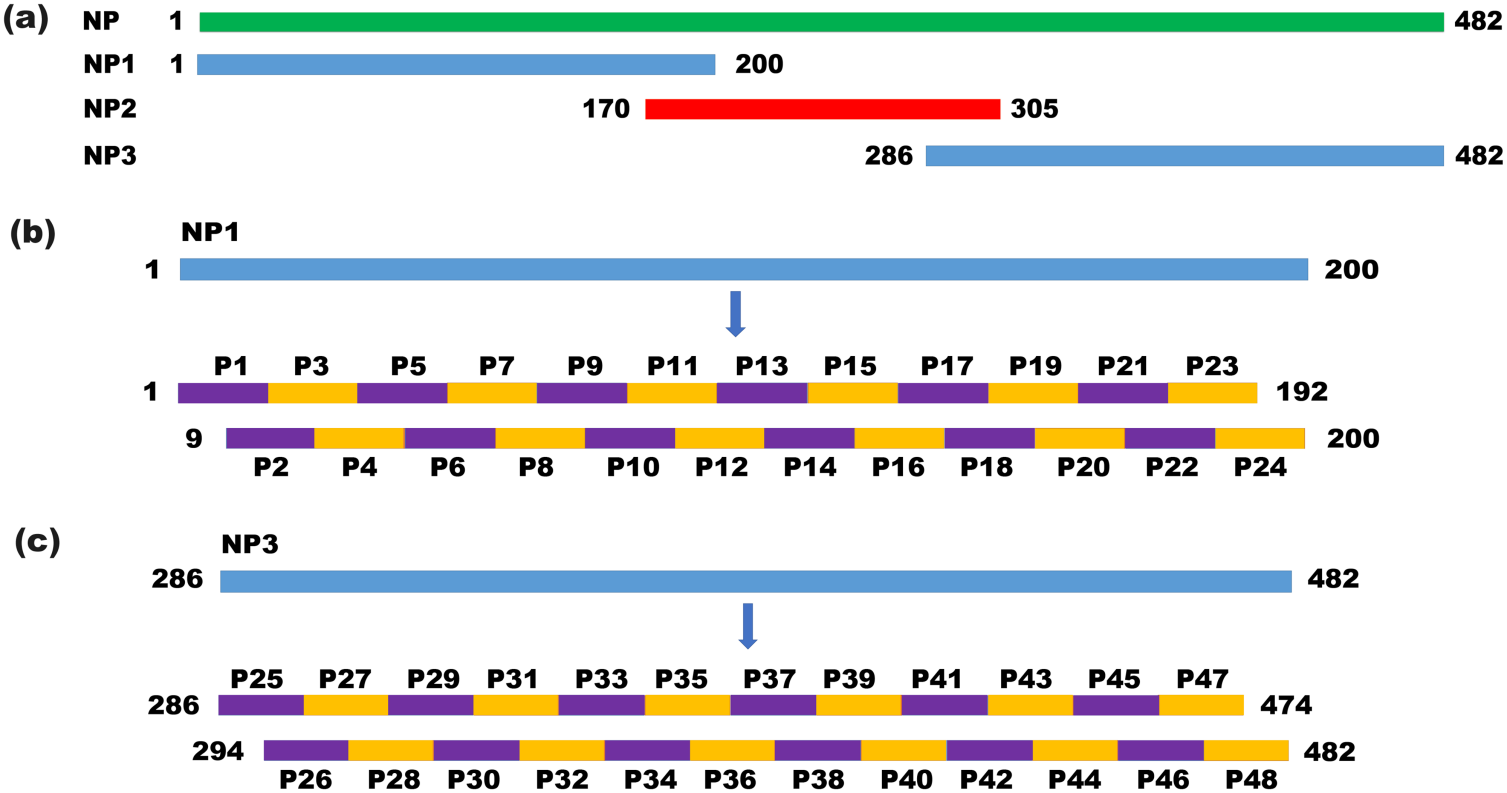
Schematic of epitope mapping strategy. The green band indicates the full length of the NP protein, the blue bands indicate the NP1 and NP3 segments and the red band represents the NP2 segment (a). Schematic of epitope mapping strategy involving 48 overlapping 16mer peptides spanning NP1 (b) and NP3 (c).

To map epitopes on the NP1 and NP3 segments, we used the feasible strategy shown in Fig 1. A total of 48 16mer peptides numbered P1-P48 were bio-expressed for the two segments NP1 and NP3. The 16mers all had an overlap of 8 aa residues between each two adjacent peptides. For fine epitope motif mapping, eight sets of a total of 58 8mer peptides (named P49-P106) with an overlap of 7 aa residues were bio-expressed based on the reactive 16mer peptides mapped in the first round of antigenic peptide mapping. The aa sequences of the biosynthesized 16/8mer peptides and their positions on NP are shown in S1 and S2 Tables, respectively.

### Construction of recombinant plasmids

All plus and minus strands of DNA fragments that encoded target 16/8mer peptides and had cohesive end nucleotides of *Bam*H I and TAA-*Sal* I sites at the 5`- and 3`- ends were synthesized by SBS Genetech Co., Ltd (Shanghai, China). Each r-plasmid expressing a 16/8mer peptide was constructed according to the GST188-BSP method [21], in which the major steps were as follows: i) conducting an annealing reaction involving paired plus and minus strands; ii) conducting a ligation reaction involving annealed DNA fragment and pXXGST-3 plasmid cut by *Bam*H I and *Sal* I; iii) transforming *E*. *coli* BL21 (DE3) competent cells with the ligation mixture; iv) screening of the r-clones by carrying out sodium dodecyl sulfate polyacrylamide gel electrophoresis (SDS-PAGE) using total proteins from each induced clone and observing whether there is a specific 16/8mer peptide on the gel; and v) sequencing of inserted DNA fragment encoding each 16/8mer peptide for each determined r-clone to ensure that all synthesized DNA sequences are accurate.

### Expression of target short peptide

Each above-determined r-clone was used to express a 16/8mer peptide in *E*. *coli* BL21 (DE3) cells, which was fused with the GST188 protein [21], that is, each r-clone was cultivated in 2 mL luria bertani (LB) medium with 100 μg/mL ampicillin at 220 rpm overnight. The following day, 30 μL of bacteria culture was added to 3 mL of fresh LB medium, grown at 30°C for 4 h to increase the bacterial density until reaching an optical density at 600 nm (OD_600_) of 0.5–0.7, and then grown at 42°C for 4 h to induce the expression of the target short peptide. All collected cell pellets containing the expressed 16/8mer peptide fusion proteins were stored at -20°C.

### SDS-PAGE and western blotting

The cell pellets obtained from 2 mL culture of expressed 16/8mer peptide were boiled at 95°C in 200 μL of 1×SDS-PAGE loading buffer for 10 min, and the proteins were resolved by 15% SDS-PAGE under reducing conditions. Two gels were obtained for each16/8mer. One gel was stained with Coomassie brilliant blue G-250 for analyzing the bands corresponding to the target 16/8mer peptide. The other gel was used for western blotting by electrotransferring the proteins onto a 0.2 μm nitrocellulose (NC) membrane [25, 26]. Regarding the specific antigen-antibody reaction, the NC membrane was blocked with 5% (w/v) skimmed milk powder in Tris-buffered saline-Tween 20 (TBS-T), treated with rabbit pAbs (1:1000 dilution) or sheep serum (1: 100 dilution) as the primary antibody, and then reacted with goat anti-rabbit IgG or mouse anti-goat IgG conjugated to HRP (1:5000 dilution) as the secondary antibody. Finally, the blot was performed using the ECL plus western blotting detection reagent according to the manufacturer’s instructions, and it was then imaged by GE-Image Quant LAS 4000 (GE Healthcare, Buckinghamshire, UK).

### Sequence alignment of homologous CCHFVs

To assess the conservation of each identified epitope among CCHFV homologous proteins, 12 NP aa sequences from different countries and genetic lineages were obtained from GenBank based on the phylogenetic tree of CCHFV strains [27]. As far as we know, 11 complete NP sequences of CCHFV strains isolated in China have been registered in the GenBank database, and these were all compared to the NP sequence investigated in this study The aa sequences of the NP1 and NP3 segments from the YL04057 strain (GenBank code: ACM78470.1) and other CCHFV-NP homologous proteins were aligned using the ClustalW program [28] and visualized using Genedoc [29].

## Results

### Antigenicity identification of NP1 and NP3 segments

**Fig 2:**
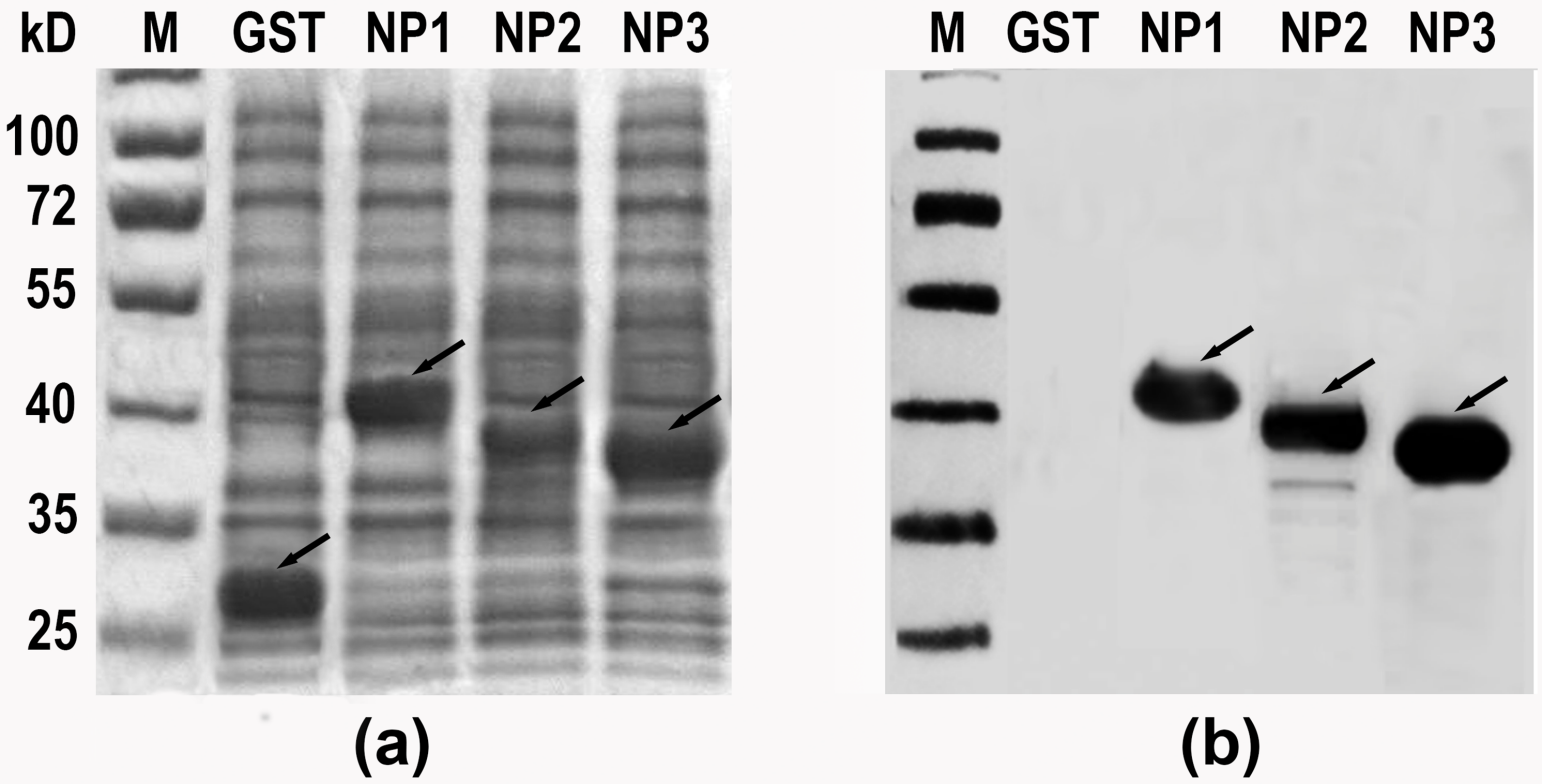
Prokaryotic expression and immunoblotting analysis of truncated NP segments. (a) SDS-PAGE analysis of expressed pET-32a-NP1, expressed pET-32a-NP3, the pGEX-KG vector (expressing GST-tagged protein as the negative control) and pGEX-KG-NP2. (b) Western blotting of NP1, NP2 and NP3 using rabbit pAbs against r-NP. The arrows represent the three expressed target segments on the gel and the reactive segments in the western blotting analysis.

In a previous study, the truncated NP1 and NP3 segments of CCHFV-NP were expressed using the prokaryotic expression vector pET-32a, and each predicted protein was fused with Trix tag, S tag and His tag (with a size about 18 kDa) [22]. However, the fusion proteins failed to be recognized by rabbit pAbs against CCHFV-NP (YL04057 strain) because of an unknown reason [22]. Numerous data on epitope mapping show that mapped epitopes do not only exist in the central area of a protein macromolecule. Therefore, to reveal all the epitopes on the NP protein after complete epitope mapping of the NP2 segment (which was cloned into a pGEX-KG vector), the predicted protein was fused with GST with a size about 26 kDa [19], the original rabbit pAbs against NP were used to re-identify the antigenicity of the NP1 and NP3 segments by immunoblotting. As shown in Fig 2, both r-segments NP1 and NP3 were recognized by the rabbit pAbs, like NP2 was, suggesting that there are also antigenic sites or epitopes on NP1 and NP3.

### Mapping of epitopes on the NP1 and NP3 segments

**Fig 3:**
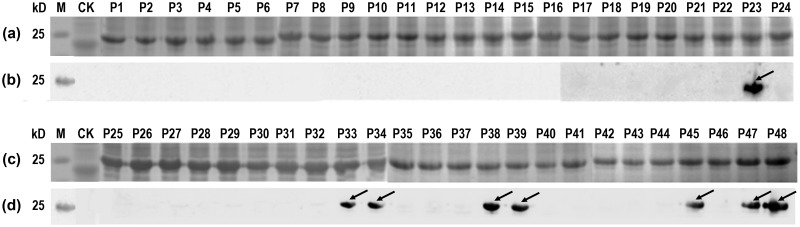
SDS-PAGE and western blotting analysis of GST188 fusion expressed 16mer peptides derived from CCHFV-NP1 and -NP3. (a, c) SDS-PAGE analysis of 48 GST188 fusion expressed 16mer peptides. (b, d) Western blot analysis of 48 GST188 fusion expressed 16mer peptides using pAbs. CK, negative control (GST188 protein expressed by pXXGST-3 vector). The arrows indicate the 16mer peptides with a positive antigen-antibody reaction in the western blotting analysis.

To determine the existence of epitopes on the NP1 and NP3 segments, both NP1 and NP3 were truncated into 24 16mer BSPs (P1-P24 and P25-P48, respectively). The 48 overlapping 16mer BSP sequences and the corresponding sites on NP are shown in S1 Table. The 48 overlapping 16mer peptides were fusion expressed with GST188 in *E*. *coli* [20], and the fusion proteins were observed as approximately 24 kDa bands on the SDS-PAGE gel (Fig 3a and 3c). In the western blotting analysis, NC membranes were blocked with 5% (w/v) skimmed milk powder, incubated sequentially with rabbit pAbs against CCHFV-NP (1:1000 dilution) and goat anti-rabbit IgG (1:5000 dilution), and then visualized by ECL. Among the 48 expression products, the following eight 16mer peptides were identified as positive by western blotting: P23, P33, P34, P38, P39, P45, P47 and P48 (Fig 3b and 3d). Of these, one 16mer peptide (P23) was located in the NP1 segment (Fig 3b), while the remaining seven 16mer peptides were located in the NP3 segment (Fig 3d). None of the 16mer peptides reacted with the CCHFV-negative rabbit sera (data not shown).

**Fig 4:**
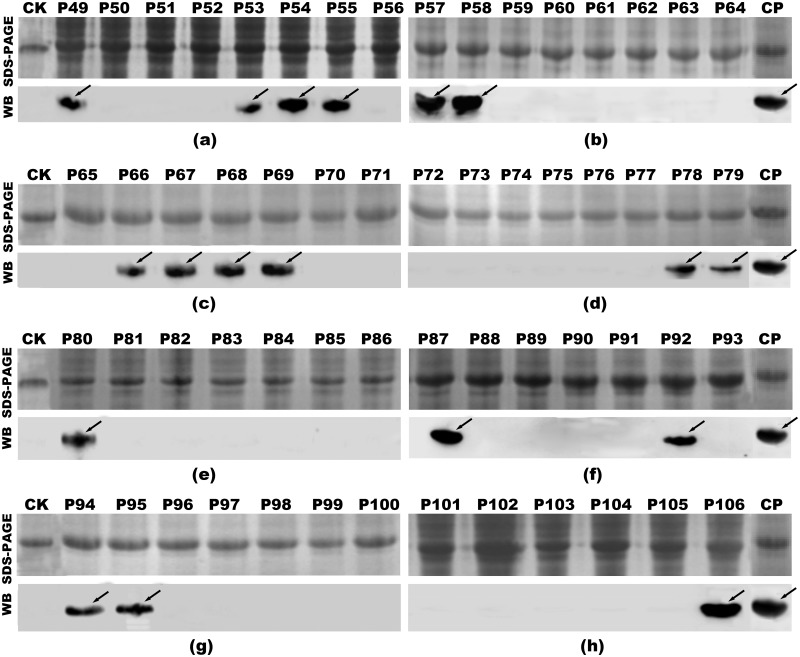
SDS-PAGE (upper layer) and western blotting analysis (lower layer) of the minimal epitopes on NP1 and NP3 using pAbs. (a) Eight 8mer peptides (P49 to P56) in 16mer peptide P23. (b) Eight 8mer peptides (P57 to P64) in 16mer peptide P33. (c) Seven 8mer peptides (P65 to P71) in 16mer peptide P34. (d) Eight 8mer peptides (P72 to P79) in 16mer peptide P38. (e) Seven 8mer peptides (P80 to P86) in 16mer peptide P39. (f) Seven 8mer peptides (P87 to P93) in 16mer peptide P45. (g) Seven 8mer peptides (P94 to P100) in 16mer peptide P47. (h) Six 8mer peptides (P101 to P106) in 16mer peptide P48. The arrows indicate the peptides with a positive antigen-antibody reaction in the western blotting analysis. CK, negative control (GST188 protein expressed by pXXGST-3); CP, positive control (16mer peptide P38 identified as positive by pAbs).

**Fig 5:**
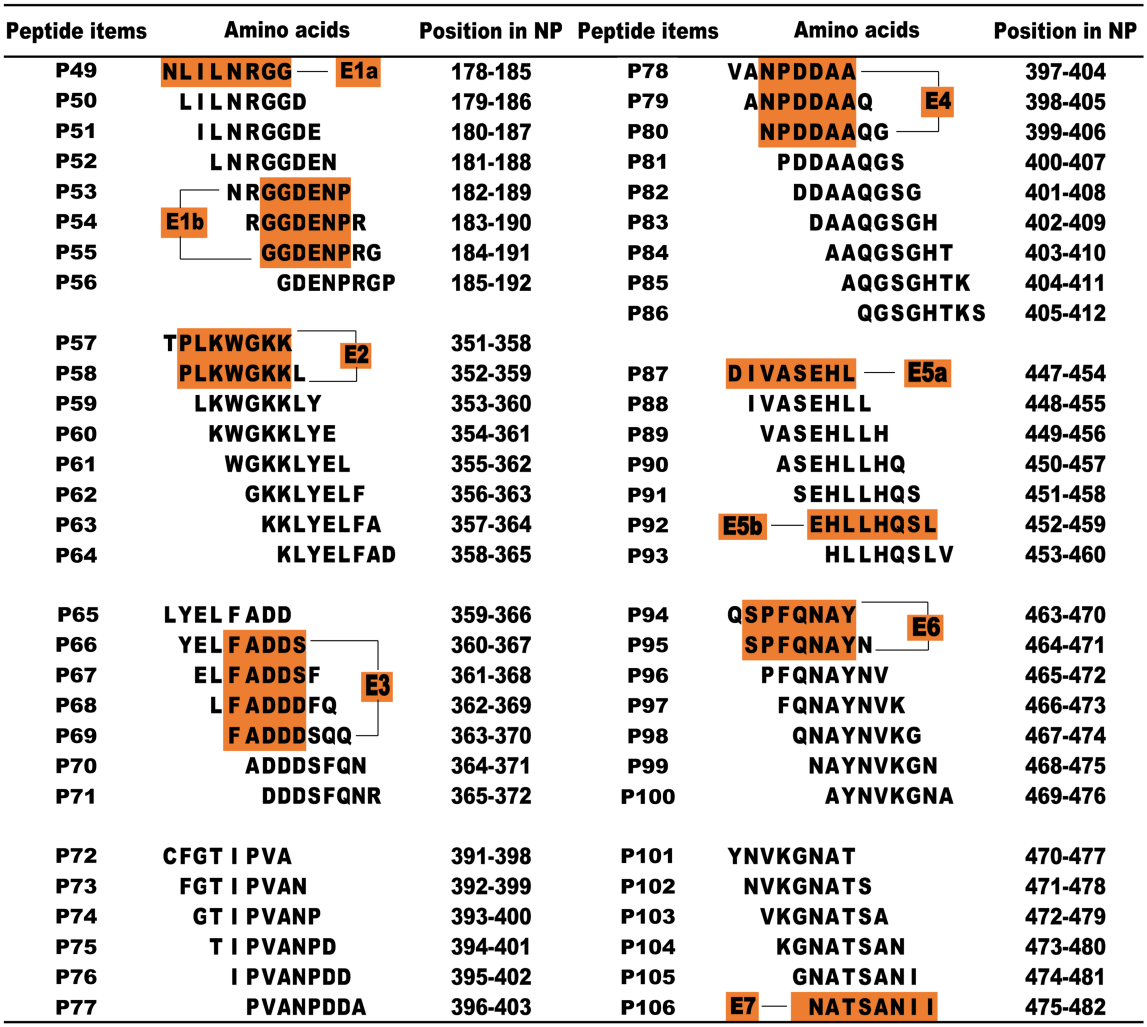
Synthetic 8mer peptide sequences derived from a span of the immunodominant peptides. The yellow-brown highlighting represents the common sequences among immunodominant peptides that react with pAbs according to western blotting analysis.

To refine and further map epitopes on NP1 and NP3, the positive 16mer peptides were screened in the next round involving 8mer peptides [30]. All 58 biosynthetic overlapping 8mer peptide aa sequences and corresponding sites on NP are shown in S2 Table. SDS-PAGE results showed that all 8mer peptides (P49-P106) with GST188 were correctly expressed in *E*. *coli* (Fig 4). In the western blotting analysis, NC membranes were blocked with 5% (w/v) skimmed milk powder, incubated sequentially with rabbit pAbs against CCHFV-NP (1:1000 dilution) and goat anti-rabbit IgG (1:5000 dilution), and then visualized by ECL. The results showed that 8mer peptides P49 (NLILNRGG), P53 (NRGGDENP), P54 (RGGDENPR) and P55 (GGDENPRG), derived from the 16mer peptide P23, were recognized by pAbs (Fig 4a). This indicates that the minimal motifs of the epitopes within P23 were ^178^NLILNRGG^185^ (designated epitope 1a, E1a) and ^184^GGDENP^189^ (E1b), based on the shared residues (Fig 5). Similarly, other antigenic peptides were further identified and analyzed (Figs 4 and 5). The fine epitopes were ^352^PLKWGKK^358^ (E2) in P33, ^363^FADDS^367^ (E3) in P34, ^399^NPDDAA^404^ (E4) in P38 and P39, ^447^DIVASEHL^454^ (E5a) and ^452^EHLLHQSL^459^ (E5b) in P45, ^464^SPFQNAY^470^ (E6) in P47, and ^475^NATSANII^482^ (E7) in P48. Thus, two specific epitopes were identified on the NP1 segment (NP^1-200^) and seven specific epitopes were identified on the NP3 segment (NP^286-482^). None of the 8mer peptides reacted with the CCHFV-negative rabbit sera (data not shown). The sensitivity of the antigen-antibody reaction was determined using the same quantity of peptides for the western blotting detection, and the data were quantitatively analyzed using Image-J software (http://rsb.info.nih.gov/ij/). The relative grayscale results showed that 8mer peptides P57, P58, P87 and P106 had a significantly (P<0.05) higher sensitivity for the antigen-antibody reaction (S1 Fig).

### Reactivity of the identified epitope motifs with anti-CCHFV serum

**Fig 6:**
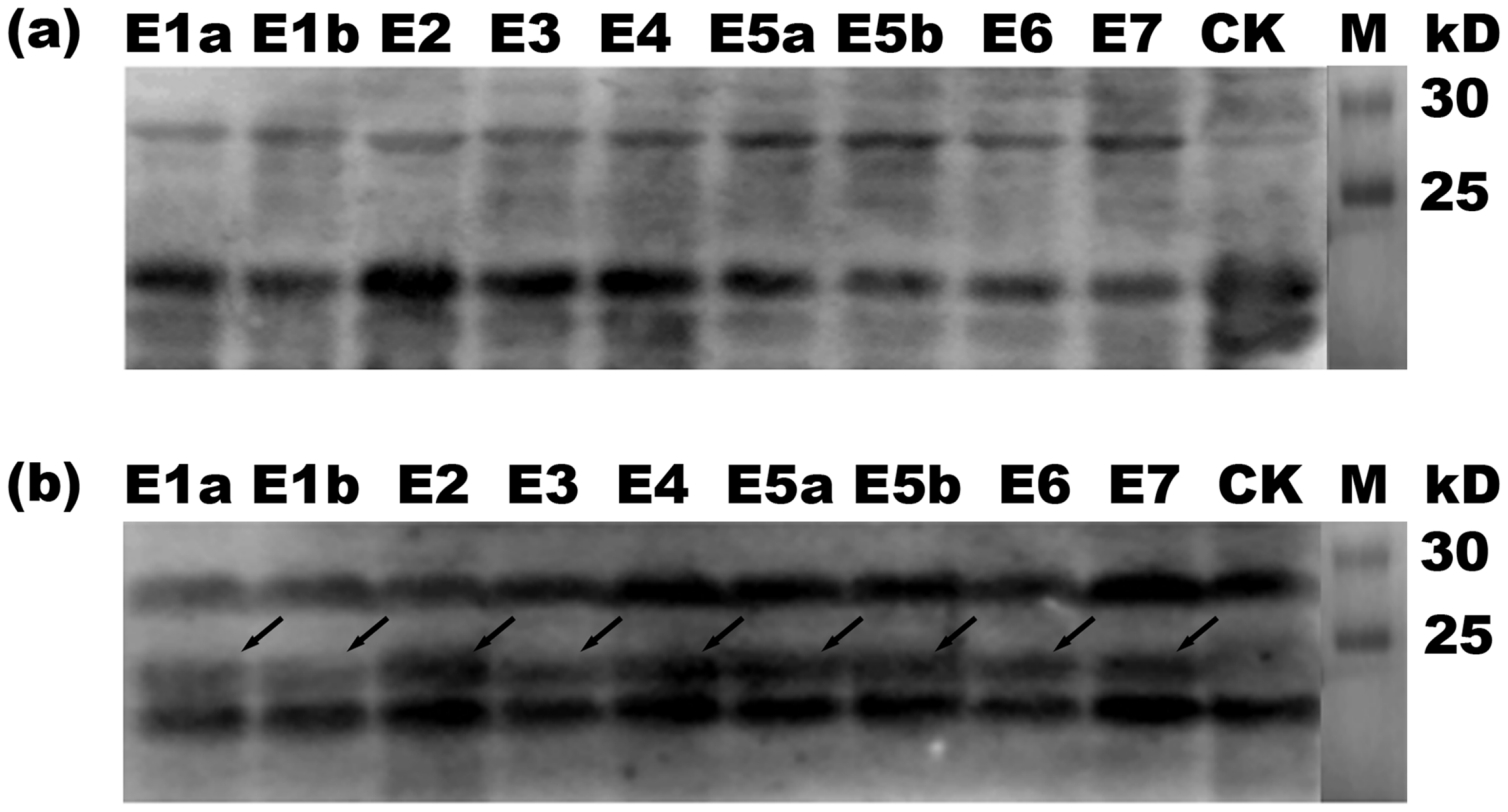
Western blotting of nine 8mer peptides containing identified epitopes performed using positive sera from sheep with a confirmed history of CCHFV infection. (a) A serum sample from healthy sheep with no history of CCHFV infection was used as a negative control. (b) A positive serum sample from a sheep with a confirmed history of CCHFV infection. The arrows represent 8mer peptides exhibiting positive antigen-antibody reactions based on western blotting analysis. CK, negative control (GST188 protein expressed by pXXGST-3).

To determine whether the minimal epitopes are rabbit specific or also recognizable by other host species, nine selected 8mer peptides, each of which contained one of the nine pAbs-identified epitopes, were subjected to western blotting using sera from sheep with or without CCHFV infection. NC membranes were blocked with 5% (w/v) skimmed milk powder, incubated sequentially with anti-CCHFV sheep serum (1:100 dilution) and mouse anti-goat IgG (1:5000 dilution), and then visualized by ECL. All the selected 8mers were recognized by the serum of sheep with a confirmed history of CCHFV infection as the primary antibody (Fig 6), while none reacted with the CCHFV antibody-negative sheep sera. Among the nine epitopes, P58 (containing E2) and P79 (containing E4) showed the strongest reaction with CCHFV antibody-positive sheep sera, whereas P49 (containing E1a) and P54 (containing E1b) displayed weaker reactions.

### 3D structures of the minimal motifs of the identified epitopes and sequence conservation analysis

**Fig 7:**
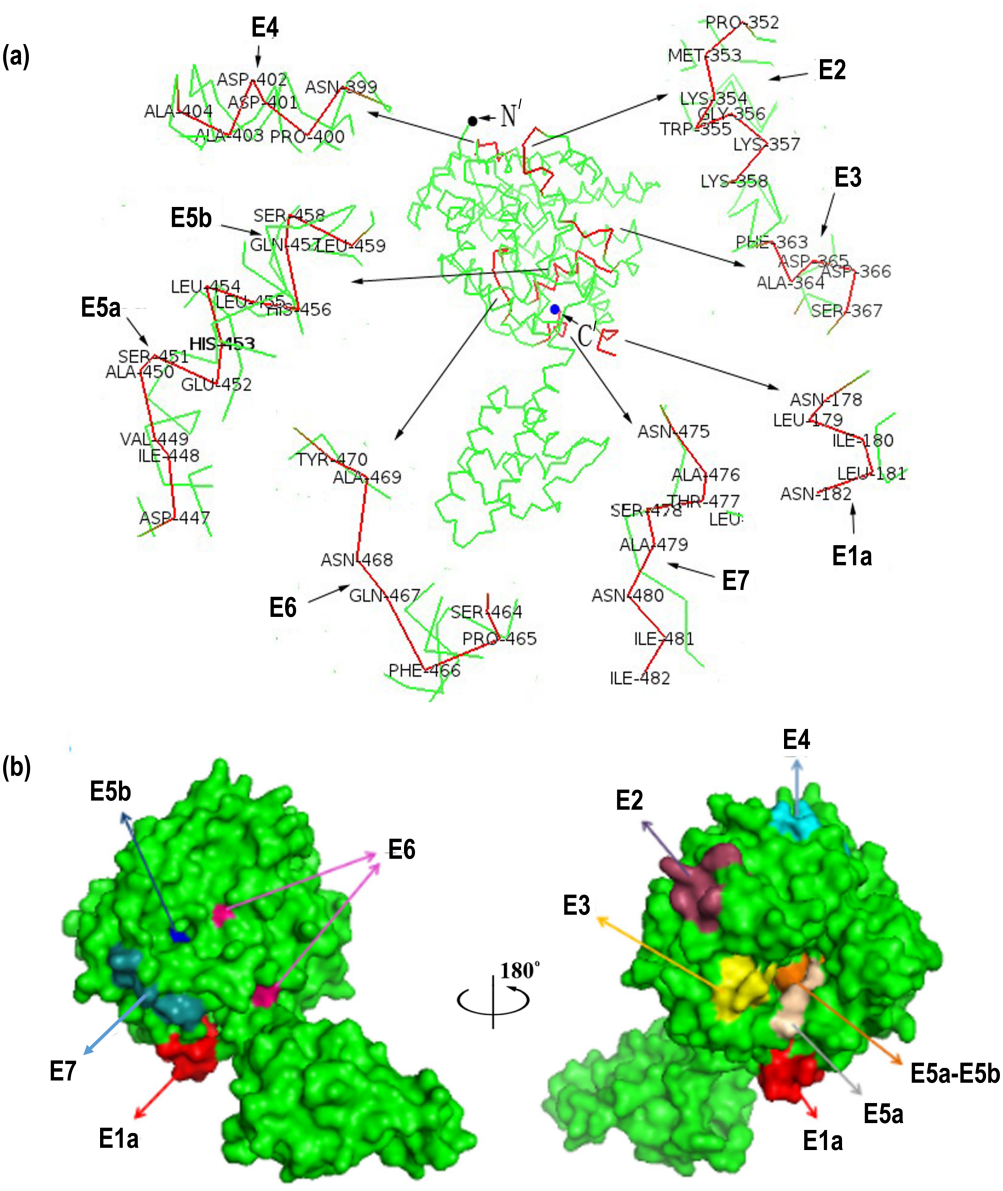
Positioning of the minimal motifs of the mapped epitopes on the predicted 3D structure of CCHFV-NP. (a) The ribbon diagram shows the overall secondary structure of CCHFV-NP from strain YL04057 (PDB code: 3U3I). The motifs within the frame indicate that the eight minimal epitopes are located on the CCHFV-NP head domain. (b) Surface properties of CCHFV-NP. The molecular surfaces of the eight minimal epitopes are shown in different colors (E1a, red; E2, magenta; E3, lemon; E4, cyan; E5a, wheat; E5a-E5b, olive; E5b, blue; E6, warm pink; E7, slate). The figures were generated using the PyMOL^™^ molecular graphics system.

PyMOL^™^ software [31] was used to simulate the 3D structure of CCHFV YL04057 NP to locate all the mapped epitopes. The results showed that E1a (^178^NLILNRGG^185^), E2 (^352^PLKWGKK^358^), E3 (^363^FADDS^367^), E4 (^399^NPDDAA^404^), E5a (^447^DIVASEHL^454^), E5b (^452^EHLLHQSL^459^), E6 (^464^SPFQNAY^470^) and E7 (^475^NATSANII^482^) are the eight epitopes that are located on the "head" surface of the NP protein (Fig 7). This is consistent with the antigenic principles of surface accessibility and hydrophilicity. In addition, eight epitopes were located on a well-defined helix-angle-helix structure (Fig 7a). As the sequence (aa 183–191) was found to be an unordered loop in the 3D structural simulation analysis, electron density was not found in the region and was not shown in the modeling. Therefore, position E1b (^184^GGDENP^189^) is not labeled in Fig 7b.

**Fig 8:**
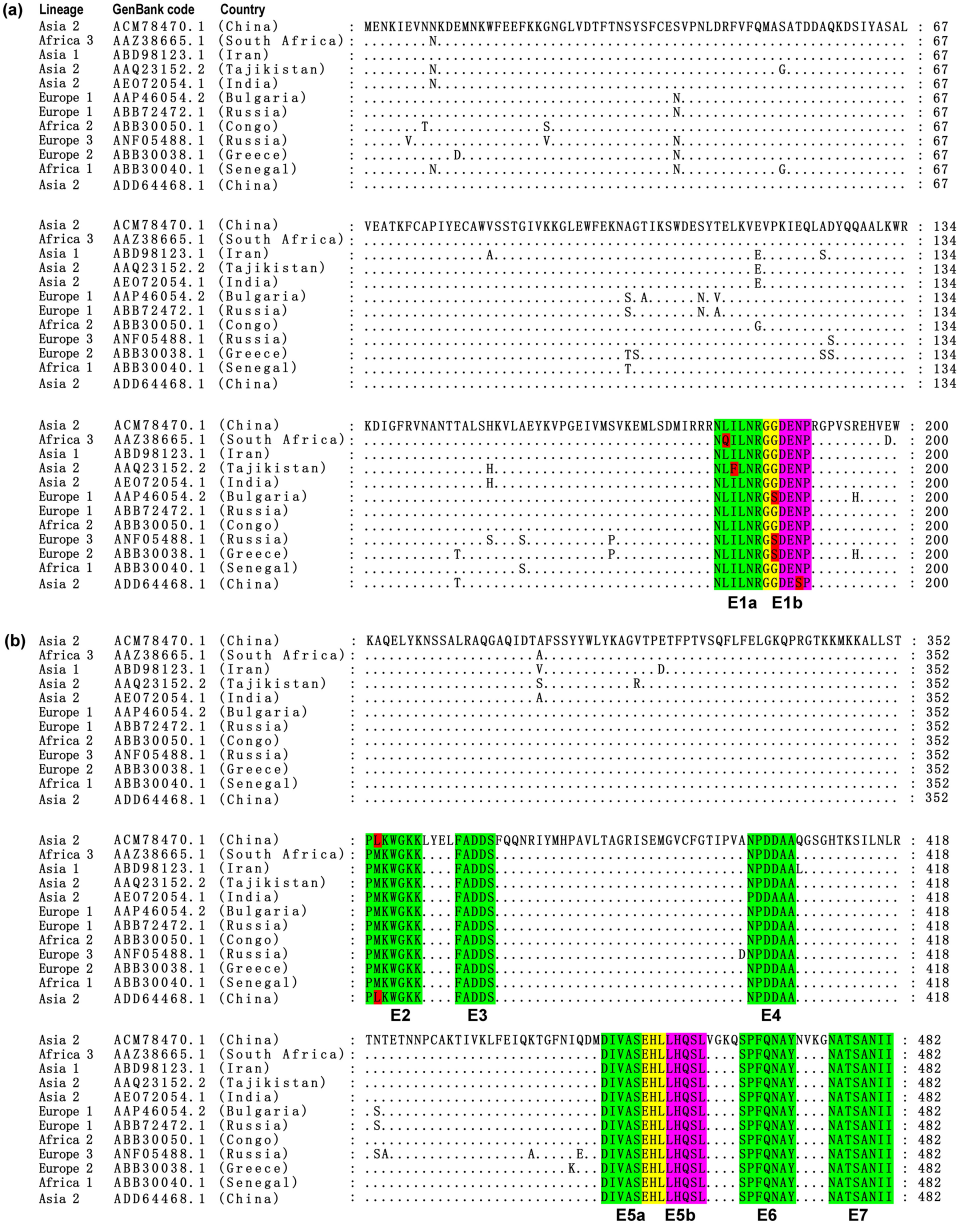
Sequence alignment of the NP1 and NP3 segments from the YL04057 strain (ACM78470.1) and other homologous CCHFV-NP proteins. (a) The aa sequence of the NP1 segment; (b) The aa sequence of the NP3 segment. The GenBank codes and sources are shown on the left. The nine minimal epitopes E1a, E1b, E2, E3, E4, E5a, E5b, E6 and E7 recognized by pAbs are highlighted, and the variable aa residues within the minimal motif epitopes are highlighted in red. Dots (.) indicate identical aa residues within all 12 strains.

To analyze the conservation of each identified BCE, multiple sequence alignment was conducted using the aa residues NP^1-200^ and NP^286-482^ from the Chinese CCHFV strain YL04057 (ACM78470.1) and 11 other strains from different countries and lineages (Fig 8). The CCHFV strains selected were representative of eight genetic lineages: Asia 1 (China, ABD98123.1), Asia 2 (Tajikistan, AAQ23152.2; India, AEO72054.1; China, ADD64468.1), Africa 1 (Senegal, ABB30040.1), Africa 2 (Congo, ABB30050.1), Africa 3 (South Africa, AAZ38665.1), Europe 1 (Bulgaria, AAP46054.2; Russia, AAB72472.1), Europe 2 (Greece, ABB30038.1) and Europe 3 (Russia, ANF05488.1). The comparison of the 12 CCHFV-NP sequences indicated that epitopes E3, E4, E5a, E5b, E6 and E7 were fully conserved. The remaining three non-conserved epitope motifs, E1a, E1b and E2, exhibited at least one aa difference when compared with their counterparts. For example, the E1a motif (^178^NLILNRGG^185^) was conserved in all CCHFV strains except the South African strain assigned to lineage Africa 3 (AAZ38665.1), in which L^179^ was substituted with Q^179^. The E1b motif (^184^GGDENP^189^) was conserved in all CCHFV strains except the Chinese strain assigned to lineage Asia 2 (ADD64468.1), in which N^188^ was substituted with S^188^. To further determine whether the epitope peptides with residue differences in Fig 8 could be used as universal diagnostic reagents, we prepared six BSPs (v_1_E1a, NQILNRGG; v_2_E1a, NLFLNRGG; v_3_E1a, NLILNRGS; v_4_E1b, GSDENP; v_5_E1b, GGDESP; and v_6_E2, PLKWGKK) and explored their antigenic properties. Specifically, single aa substitutions were made within E1a (L^179^Q, I^180^F and G^185^S), E1b (G^185^S and N^188^S) and E2 (L^353^M). The results show that the two BSPs with one variable residue v_1_E1a (L^179^Q) and v_5_E1b (N^188^S) strongly reacted with rabbit pAbs against CCHFV-NP, but the others had no reactivity (S2 Fig). This indicated that BCEs E1a, E1b and E2 derived from different CCHFV strains were not fully conserved regarding their antigenicity. I^180^, G^185^ and L^353^ may be the critical residues for antigen-antibody reactions in E1a, E1b and E2, respectively. Compared with the 11 CCHFV strains from China, the similarity of epitope E1a was 95.00%, the similarity of E1b was 93.33% and the similarity of E2 was 96.10%. Epitopes E3, E4, E5a, E5b, E6 and E7 were fully conserved (S3 Fig). Therefore, these nine epitopes can be used as candidate antigen peptides in CCHFV general diagnostic and vaccine studies.

## Discussion

CCHFV-NP is the major viral protein that can be detected in the early stages of CCHFV infection. Serological studies have shown that patients have an early and high titer of antibody response to NP [32]. Due to its highly conserved features, CCHFV-NP can be used to develop diagnostic antigens and vaccines [19]. For rational vaccine design or development of diagnostic tools, it is necessary to map the specific and conserved BCEs of target proteins [21]. Also, multi-epitope-based vaccines offer numerous advantages compared to the complete protein, including cost-effective production, stability under different conditions and multivalency [24]. Recently, Zhao et al. [33] constructed a recombinant epitope vaccine that contains six immunodominant epitopes, and they demonstrated its capacity to induce a robust IgG specific response. However, problems remain regarding incorporating more selectable BCEs from a given antigen. Therefore, it is crucial to reveal all BCEs on a target antigen for the development of an effective multivalent peptide vaccine in future.

Saijo et al. [15] previously reported that high titer antisera from CCHF patients can react with NP^201-306^ intermediate segments, which are highly conserved in many CCHFV strains. Previously, Wei et al. [22] also found that NP^237-305^ was a dominant antigenic region. Burt et al. [34] identified the antigenicity of the region N^182-195^, and Liu et al. [19] performed a fine IgG-epitope analysis of the CCHFV-NP2 segment (NP^237-305^). Although these studies confirm that the central region of CCHFV-NP is highly antigenic, the N- (NP^1-200^) and C-terminals (NP^286-482^) of CCHFV-NP also have antigenicity (Fig 2). However, there are no previous reports of epitope identification in the 1–181 and 306–486 regions of CCHFV-NP. Carter et al. [10] found that the N- and C-terminals of the head region of CCHFV-NP were involved in RNA binding. This region may play an important role in the replication of the virus or the activation of the host’s early humoral immunity [12].

Epitope mapping is the key to basic and applied research in the field of virology [35]. Compared to other techniques such as r-DNA [36], peptide synthesis [37], and peptide/protein display technology [38], the BSP method is feasible and has been successfully used in the identification of certain target antigen epitopes, such as the epitopes of human egg zona pellucida protein [20], the nucleoprotein of Peste des petits ruminants virus (PPRV) [35], and the E6, E7 and L1 proteins of human papillomavirus type 58 [30]. The BSP method is commonly used to expressmultiple polypeptide segments of 8–20 aa residues, which cover all of a target protein [21]. This further confirms that the BSP method is a reliable epitope scanning mapping strategy.

In this paper, to carry out a comprehensive linear epitope scan of CCHFV-NP, the modified epitopes of N-terminal NP1 and C-terminal NP3 segments were identified using a modified BSP method. First, we successfully synthesized 48 overlapping 16mer peptides and 58 overlapping 8mer peptides, and we identified nine minimal BCE linear motifs by western blotting with pAbs. The epitopes comprised E1a (^178^NLILNRGG^185^), E1b (^184^GGDENP^189^), E2 (^352^PLKWGKK^358^), E3 (^363^FADDS^367^), E4 (^399^NPDDAA^404^), E5a (^447^DIVASEHL^454^), E5b (^452^EHLLHQSL^459^), E6 (^464^SPFQNAY^470^) and E7 (^475^NATSANII^482^).

The sequences of 12 strains of CCHFV from different countries and lineages were analyzed. The results showed that six epitopes, E3, E4, E5a, E5b, E6 and E7, were 100% conserved among the 12 CCHFV strains, while the epitopes of E1a, E1b and E2 showed homologies of 93.75%, 96.67% and 97.14%, respectively. Burt and colleagues [34] predicted the antigen epitopes of CCHFV-NP and synthesized more than 60 peptides according to the results of a software analysis. Thereafter, the antigenicity of these peptides was tested with patient sera using enzyme-linked immunosorbent assay (ELISA). Their results showed that the "NRGGDENPRGPVSR" polypeptide at the aa residue position of NP^182-195^ reacted with 13 of 16 human serum samples and had high antigenicity. Our study identified the epitope E1b (^184^GGDENP^189^), which is consistent with results from Burt et al [34].

The NP 3D structure and the exact sites of the nine smallest epitope motifs of CCHFV-NP were analyzed using PyMOL^™^ software. The results showed that only E1b (^184^GGDENP^189^) is located on unordered loop with no electron density, according to the 3D structural simulation analysis. The other eight motifs were exposed on the surface of the 3D structure, in the helix-angle-helix region (Fig 7b), indicating that they could easily bind to antibodies. The epitopes located on the surface of the target protein play an important role in the future development of drugs that interact with target antigens. Wadood and colleagues [39] first designed epitope-based drugs related to the dengue virus envelope protein and then successfully screened for drugs that interact with viral epitopes. Their results provide novel and potential drugs for the treatment of dengue fever.

Epitopes identified in this study are likely to be highly species-specific. Epitopes E3, E4, E5a, E5b, E6 and E7 in the NP3 segment of 12 strains from different lineages (Fig 8) and 11 strains from China were highly conserved, with a similarity of 100% (S3 Fig), indicating that the six epitopes on the NP3 segment may be conserved epitope sequences of CCHFV. Whether the minimal antigenic epitopes identified in this study can induce the long-term production of neutralizing antibodies requires further studies, as CCHFV is classified as a BSL4 pathogen. This indicates that CCHFV may cause serious public health threats. Studies on the identification of neutralizing antibodies and other experimental studies of CCHFV are limited [40]. Therefore, future work also needs to establish suitable cell and animal models to further identify the neutralizing activity related to these epitopes.

In conclusion, nine fine epitope motifs were obtained and analyzed for antigenicity, which provided new data for the development of a CCHFV multi-epitope detection kit and a multi-epitope peptide vaccine. The results also provide a theoretical basis for the elucidation of CCHFV pathogenesis and immune defense mechanisms.

## Supporting information

S1 Table 16mer peptide amino acid sequences and their location on CCHFV YL04057 NP. (DOC)

S2 Table 8mer peptide amino acid sequences and their location on CCHFV YL04057 NP. (DOC)

S1 Fig Relative grayscale level analyses of 8mer peptides from western blotting using Image-J software. To determine the sensitivity of the antigen-antibody reaction involving the 8mer peptides, quantitative analyses were performed using the same quantity of peptides for detection. The relative grayscale level of each 8mer peptide compared to the positive control, CP (16mer peptide P38, identified as positive by pAbs), was analyzed according to the results in Fig 4. Statistical analysis of data was performed using one-way analysis of variance (ANOVA) to determine the significant differences using SPSS software. Letters (a, b, c) indicate the significant differences (P<0.05).
(TIF)

S2 Fig SDS-PAGE and western blotting analysis of mutated peptides derived from epitopes E1a, E1b and E2. According to the results of homologous analysis in Fig 8, substitutions at L^179^Q, I^180^F and G^185^S in E1a, G^185^S and N^188^S in E1b, and L^353^M in E2 were mutated to analyze the antigenicity. CK, negative control (GST188 protein expressed by pXXGST-3).
(TIF)

S3 Fig Analysis of conservation of mapped epitopes among 11 CCHFV strains. As far as we know, 11 complete nucleocapsid protein (NP) sequences of CCHFV strains isolated in China have been registered in the GenBank database. The 11 Chinese strains’ sequences corresponding to aa residues 1 to 200 of NP (Figure A in S3 Fig) and 286 to 482 of NP (Figure B in S3 Fig) were retrieved from GenBank for sequence alignment using the ClustalW program. The 11 strains showed good conservation at the E2, E3, F4, E5a, E5b, E6 and E7 sites. There was only differences in E1a (R^183^S), E1b (N^188^S) and E2 (L^353^M). GenBank code ACM78470.1 represents the CCHFV YL04057 strain.
(TIF)
