# Supplementary material for: Mapping of B-cell epitopes on the N- terminal and C-terminal segment of nucleocapsid protein from Crimean-Congo hemorrhagic fever virus
Source: PLoS One. 2018 Sep 20;13(9):e0204264. doi: 10.1371/journal.pone.0204264 (PMC6147494; doi:10.1371/journal.pone.0204264)
Supplement: S1 Table — (DOC) [file pone.0204264.s001.doc]

**S1 table.** **16mer peptides amino acid sequence and its location on CCHFV YL04057 NP**.

| Peptide items | Position in NP | Amino acids | Peptide items | Position in NP | Amino acids |
| --- | --- | --- | --- | --- | --- |
| P1 | **NP^1-16^** | MENKIEVNNKDEMNKW | P25 | **NP^286-301^** | KAQELYKNSSALRAQG |
| P2 | **NP^9-24^** | NKDEMNKWFEEFKKGN | P26 | **NP^294-309^** | SSALRAQGAQIDTAFS |
| P3 | **NP^17-32^** | FEEFKKGNGLVDTFTN | P27 | **NP^302-317^** | AQIDTAFSSYYWLYKA |
| P4 | **NP^25-40^** | GLVDTFTNSYSFCESV | P28 | **NP^310-325^** | SYYWLYKAGVTPETFP |
| P5 | **NP^33-48^** | SYSFCESVPNLDRFVF | P29 | **NP^318-333^** | GVTPETFPTVSQFLFE |
| P6 | **NP^41-56^** | PNLDRFVFQMASATDD | P30 | **NP^326-341^** | TVSQFLFELGKQPRGT |
| P7 | **NP^49-64^** | QMASATDDAQKDSIYA | P31 | **NP^334-349^** | LGKQPRGTKKMKKALL |
| P8 | **NP^57-72^** | AQKDSIYASALVEATK | P32 | **NP^342-357^** | KKMKKALLSTPLKWGK |
| P9 | **NP^65-80^** | SALVEATKFCAPIYEC | P33 | **NP^350-365^** | STPLKWGKKLYELFAD |
| P10 | **NP^73-88^** | FCAPIYECAWVSSTGI | P34 | **NP^358-373^** | KLYELFADDSFQQNRI |
| P11 | **NP^81-96^** | AWVSSTGIVKKGLEWF | P35 | **NP^366-381^** | DSFQQNRIYMHPAVLT |
| P12 | **NP^89-104^** | VKKGLEWFEKNAGTIK | P36 | **NP^374-389^** | YMHPAVLTAGRISEMG |
| P13 | **NP^97-112^** | EKNAGTIKSWDESYTE | P37 | **NP^382-397^** | AGRISEMGVCFGTIPV |
| P14 | **NP^105-120^** | SWDESYTELKVEVPKI | P38 | **NP^390-405^** | VCFGTIPVANPDDAAQ |
| P15 | **NP^113-128^** | LKVEVPKIEQLADYQQ | P39 | **NP^398-413^** | ANPDDAAQGSGHTKSI |
| P16 | **NP^121-136^** | EQLADYQQAALKWRKD | P40 | **NP^406-421^** | GSGHTKSILNLRTNTE |
| P17 | **NP^129-144^** | AALKWRKDIGFRVNAN | P41 | **NP^414-429^** | LNLRTNTETNNPCAKT |
| P18 | **NP^137-152^** | IGFRVNANTTALSHKV | P42 | **NP^422-437^** | TNNPCAKTIVKLFEIQ |
| P19 | **NP^145-160^** | TTALSHKVLAEYKVPG | P43 | **NP^430-445^** | IVKLFEIQKTGFNIQD |
| P20 | **NP^153-168^** | LAEYKVPGEIVMSVKE | P44 | **NP^438-453^** | KTGFNIQDMDIVASEH |
| P21 | **NP^161-176^** | EIVMSVKEMLSDMIRR | P45 | **NP^446-461^** | MDIVASEHLLHQSLVG |
| P22 | **NP^169-184^** | MLSDMIRRRNLILNRG | P46 | **NP^454-469^** | LLHQSLVGKQSPFQNA |
| P23 | **NP^177-192^** | RNLILNRGGDENPRGP | P47 | **NP^462-477^** | KQSPFQNAYNVKGNAT |
| P24 | **NP^185-200^** | GDENPRGPVSREHVEW | P48 | **NP^470-482^** | YNVKGNATSANII |
