# Supplementary material for: Mapping of B-cell epitopes on the N- terminal and C-terminal segment of nucleocapsid protein from Crimean-Congo hemorrhagic fever virus
Source: PLoS One. 2018 Sep 20;13(9):e0204264. doi: 10.1371/journal.pone.0204264 (PMC6147494; doi:10.1371/journal.pone.0204264)
Supplement: S2 Table — (DOC) [file pone.0204264.s002.doc]

| **S2 table. 8mer peptides amino acid sequence and its location on CCHFV YL04057 NP.** | | | | | | | | |
| --- | --- | --- | --- | --- | --- | --- | --- | --- |
| positive 16mer peptides | P23 | P33 | P34 | P38 | P39 | P45 | P47 | P48 |
|  | NP^177-192^ | NP3^50-365^ | NP3^58-373^ | NP^390-405^ | NP^398-413^ | NP^446-461^ | NP^462-477^ | NP^470-482^ |
| Corresponding 8mer peptides | NLILNRGG | TPLKWGKK | LYELFADD | CFGTIPVA | NPDDAAQG | DIVASEHL | QSPFQNAY | YNVKGNAT |
|  | LILNRGGD | PLKWGKKL | YELFADDS | FGTIPVAN | PDDAAQGS | IVASEHLL | SPFQNAYN | NVKGNATS |
|  | ILNRGGDE | LKWGKKLY | ELFADDSF | GTIPVANP | DDAAQGSG | VASEHLLH | PFQNAYNV | VKGNATSA |
|  | LNRGGDEN | KWGKKLYE | LFADDSFQ | TIPVANPD | DAAQGSGH | ASEHLLHQ | FQNAYNVK | KGNATSAN |
|  | NRGGDENP | WGKKLYEL | FADDSFQQ | IPVANPDD | AAQGSGHT | SEHLLHQS | QNAYNVKG | GNATSANI |
|  | RGGDENPR | GKKLYELF | ADDSFQQN | PVANPDDA | AQGSGHTK | EHLLHQSL | NAYNVKGN | NATSANII |
|  | GGDENPRG | KKLYELFA | DDSFQQNR | VANPDDAA | QGSGHTKS | HLLHQSLV | AYNVKGNA |  |
|  | GDENPRGP | KLYELFAD |  | ANPDDAAQ |  |  |  |  |
