# Supplementary figures and images for: Mapping of B-cell epitopes on the N- terminal and C-terminal segment of nucleocapsid protein from Crimean-Congo hemorrhagic fever virus
Source: PLoS One. 2018 Sep 20;13(9):e0204264. doi: 10.1371/journal.pone.0204264 (PMC6147494; doi:10.1371/journal.pone.0204264)

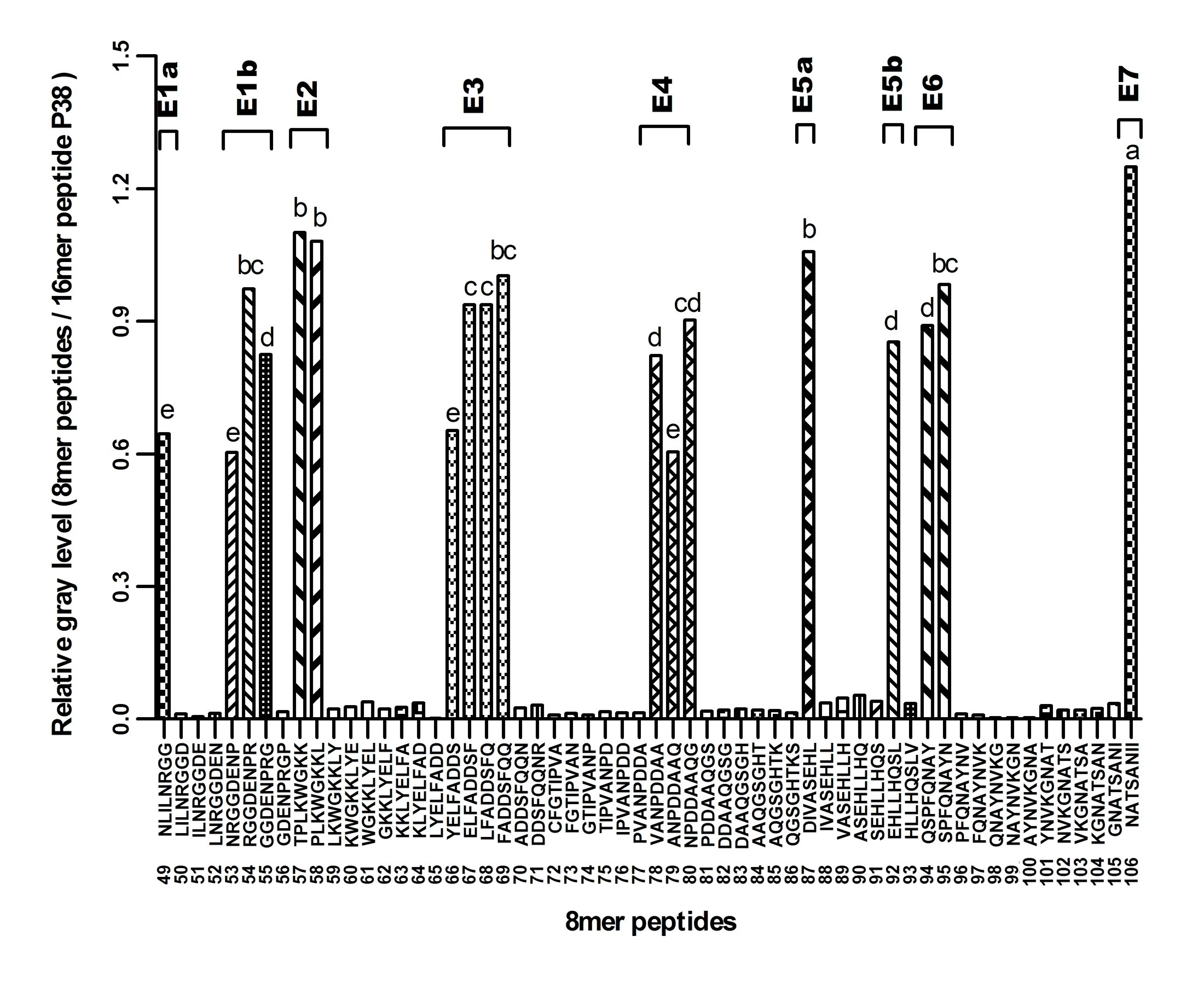

Supplement: S1 Fig — To determine the sensitivity of the antigen-antibody reaction involving the 8mer peptides, quantitative analyses were performed using the same quantity of peptides for detection. The relative grayscale level of each 8mer peptide compared to the positive control, CP (16mer peptide P38, identified as positive by pAbs), was analyzed according to the results in Fig 4. Statistical analysis of data was performed using one-way analysis of variance (ANOVA) to determine the significant differences using SPSS software. Letters (a, b, c) indicate the significant differences (P<0.05). (TIF) [file pone.0204264.s003.tif]

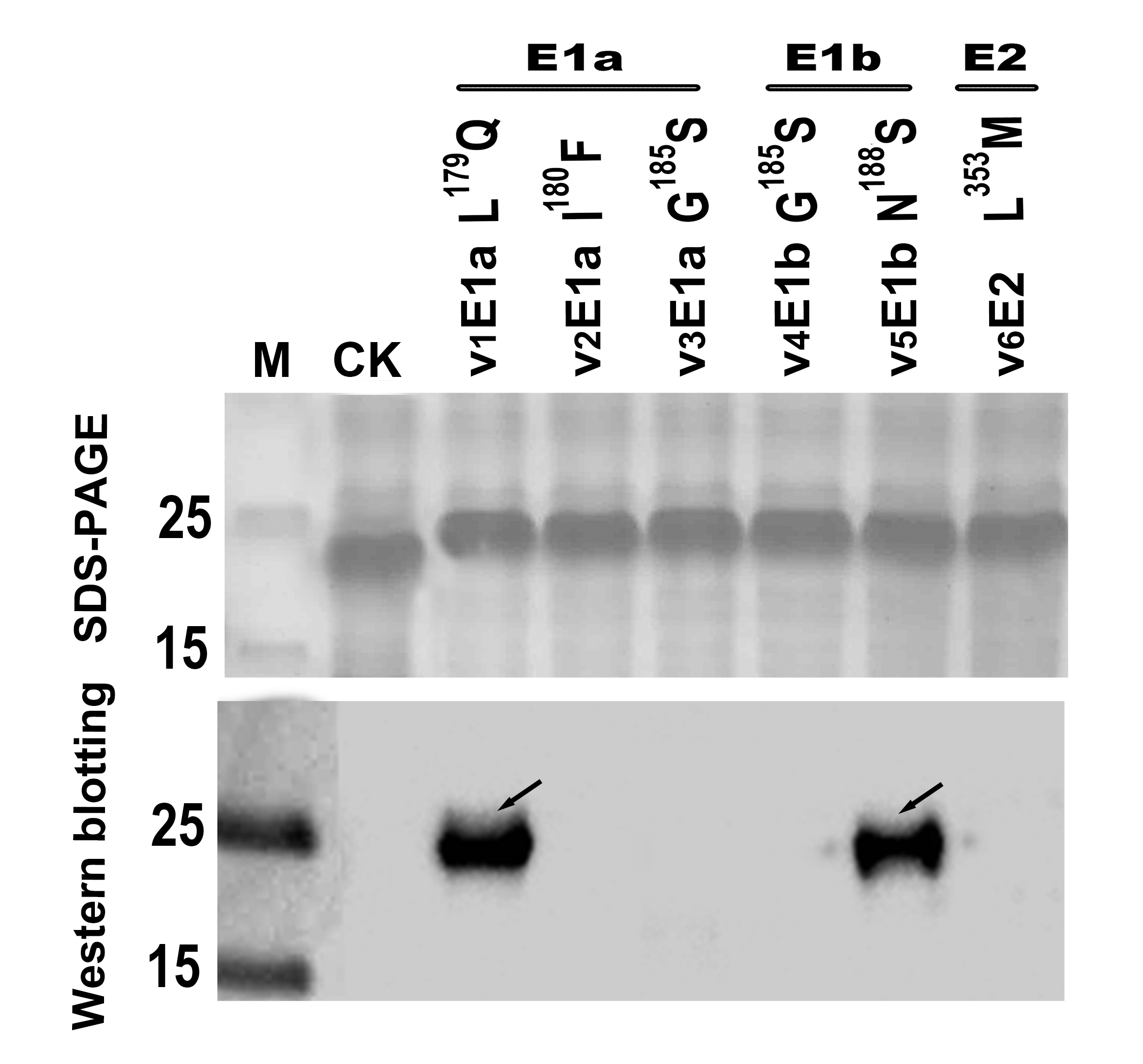

Supplement: S2 Fig — According to the results of homologous analysis in Fig 8, substitutions at L179Q, I180F and G185S in E1a, G185S and N188S in E1b, and L353M in E2 were mutated to analyze the antigenicity. CK, negative control (GST188 protein expressed by pXXGST-3). (TIF) [file pone.0204264.s004.tif]

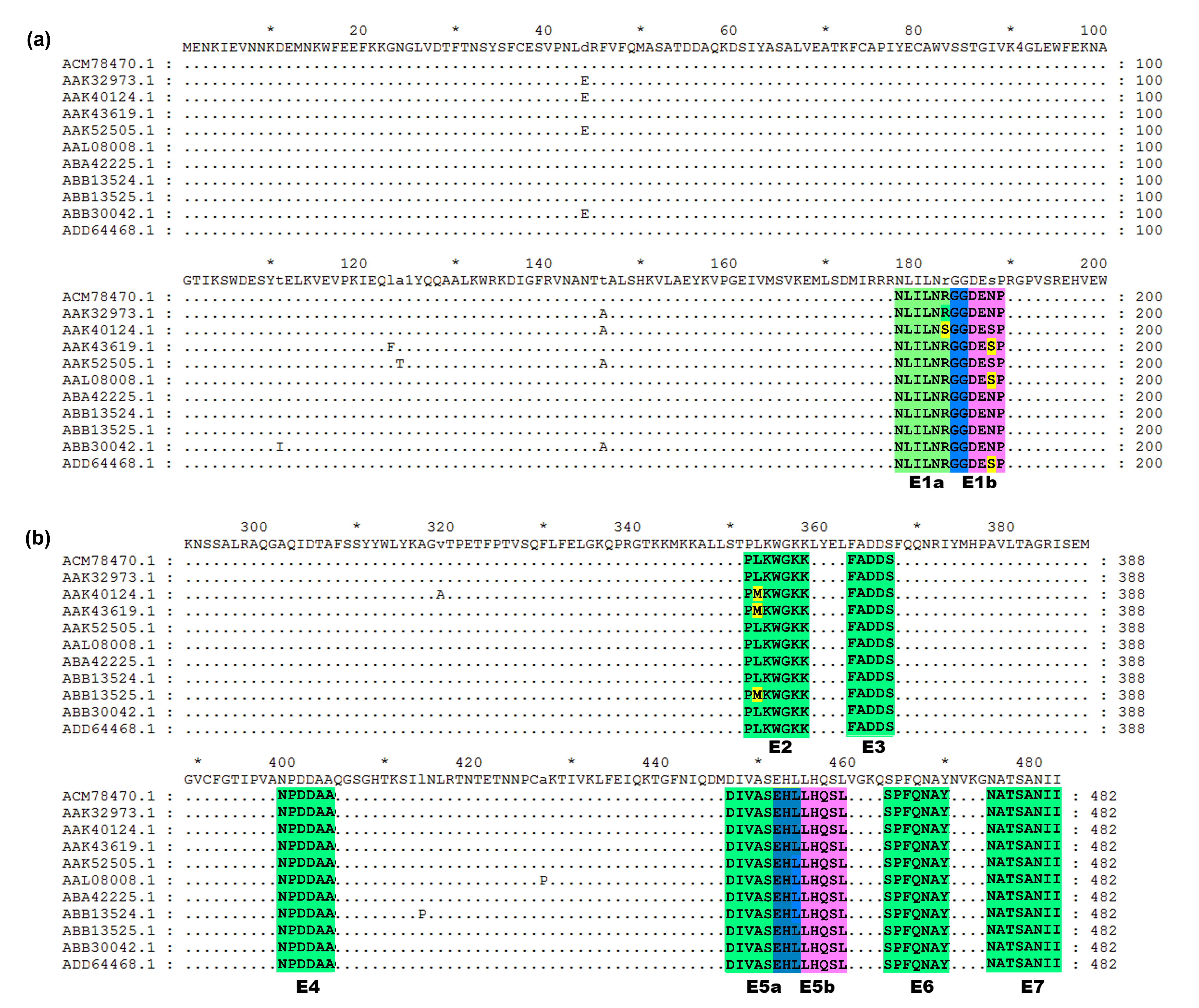

Supplement: S3 Fig — As far as we know, 11 complete nucleocapsid protein (NP) sequences of CCHFV strains isolated in China have been registered in the GenBank database. The 11 Chinese strains’ sequences corresponding to aa residues 1 to 200 of NP (Figure A in S3 Fig) and 286 to 482 of NP (Figure B in S3 Fig) were retrieved from GenBank for sequence alignment using the ClustalW program. The 11 strains showed good conservation at the E2, E3, F4, E5a, E5b, E6 and E7 sites. There was only differences in E1a (R183S), E1b (N188S) and E2 (L353M). GenBank code ACM78470.1 represents the CCHFV YL04057 strain. (TIF) [file pone.0204264.s005.tif]
